# Intralymphatic immunotherapy induces allergen specific plasmablasts and increases tolerance to skin prick testing in a pilot study

**DOI:** 10.1186/s13601-016-0107-x

**Published:** 2016-05-25

**Authors:** Johannes Martin Schmid, Homaira Nezam, Hans Henrik Torp Madsen, Alexander Schmitz, Hans Jürgen Hoffmann

**Affiliations:** Department of Respiratory Diseases and Allergy, Aarhus University Hospital, Norrebrogade 44, 8000 Aarhus C, Denmark; Department of Clinical Medicine, Aarhus University, Aarhus, Denmark; Department of Diagnostic Radiology, Aarhus University Hospital, Aarhus, Denmark; Department of Haematology, Medical Center, Aalborg Hospital Science and Innovation Center AHSIC, Aalborg University, 9000 Aalborg, Denmark

**Keywords:** Allergen immunotherapy, Intralymphatic immunotherapy, Grass pollen allergy, Allergen specific plasmablasts, Symptom medication score

## Abstract

**Background:**

Allergen Immunotherapy is a promising treatment of allergy. Seven patients with rhinoconjunctivitis to grass allergen were treated with intralymphatic immunotherapy (ILIT) to explore whether this treatment could be performed. Effect of treatment was assessed as change in symptom medication score, response in skin prick test and nasal allergen provocation. ILIT deposits allergen in an inguinal lymph node to elicit a strong immune stimulus. This allowed us to monitor appearance of allergen specific plasmablasts 7 days after allergen injection.

**Findings:**

In an open trial of seven patients with a history of symptomatic allergic rhinoconjunctivitis due to grass pollen, three injections of allergen into inguinal lymph nodes were performed with monthly intervals. Allergen injections induced grass allergen specific plasmablasts expressing other isotypes than IgE after 7 days, induced a trend toward improvement in symptom and medication score and rhinoconjunctivitis-related quality of life during the grass pollen season 2013 and significantly raised the threshold in nasal allergen challenge and titrated skin prick testing. Mild side-effects were recorded after 3 of the 21 of injections (14 %).

**Conclusions:**

This pilot study shows that ILIT may induce allergen specific plasmablasts, and confirms an effect on provocation of mast cells in skin and nasal mucosa during the ensuing winter.

## Background

Allergic rhinoconjunctivitis is a growing global health problem and causes a significant clinical and socio-economic burden in Western countries [1]. English high school graduates with allergic airway disease finish school with a lower grade than non-allergic high school graduates [2]. The only disease modifying treatment available is allergen immunotherapy (AIT), which targets the underlying immunological mechanisms. Subcutaneous immunotherapy (SCIT) is a time demanding and costly treatment, requiring 30–80 injections over 3–5 years. Sublingual immunotherapy requires long-term treatment where compliance may be an issue [3].

The efficacy of intralymphatic immunotherapy (ILIT), where allergen is injected into inguinal lymph nodes, has been assessed in recent studies [4–6]. Two trials published reported clinical effect comparable to that of SCIT [4, 5]. A recent placebo-controlled randomized trial with injections every 2 weeks had a large number of side effects and did not show clinical effect [6]. We would like to contribute to this discussion with our open pilot study. We involved a radiologist to ascertain that we injected allergen into lymph nodes as this may be paramount to the success of this treatment [7] and adhered to the protocol of former, successful trials in which injections of 1000 SQU were given at more than 4-weekly intervals [4, 5, 8].

AIT is thought to induce a protective IgG response [9]. Circulating plasmablasts are required for induction of new immunoglobulin, and have previously been identified in circulation 7 days after a tetanus vaccination [10]. We reasoned that we would be able to identify allergen specific plasma blasts in circulation 7 days after injection of allergen in an inguinal lymph node.

The aim of this pilot project was to establish ILIT in our clinic and to evaluate clinical and paraclinical outcome measures.

## Methods

Seven patients (age range 23–41, 4 women) with a history of symptomatic rhino-conjunctivitis due to grass pollen allergy recruited as open control group for a SCIT study [9] accepted the option of ILIT as alternative to SCIT. Diagnosis was confirmed by positive skin prick test (SPT, wheal >3 mm) and positive nasal allergen challenge (NAC). The regional scientific ethics committee approved the study (Number 1-10-72-132-13, 13-06-2013). It was monitored by the local GCP unit and was conducted in accordance with good clinical practice guidelines. All participants gave written informed consent before the study was initiated. This study is registered in Eudra-CT with number 2012-005227-33, 22-03-2013.

Allergen extract (0.1 ml Alutard Phleum pratense, 10.000 SQU/ml, ALK-abelló, Hoersholm, Denmark) was injected into a superficial inguinal lymph node guided by ultrasound (GE Logiq S8). Injections were given on the same side and preferably into the same lymph node with 23–36 days interval. The last injection was given 2 weeks after initiation of the 2013 grass pollen season. Participants recorded daily combined symptom and medication score (SMS) and weekly rhninitis related quality of life using the RQLQ(s) questionnaire during the grass pollen seasons of 2012 and 2013. Patients 2 and 4 did not submit SMS data. Daily pollen counts were obtained from the Danish Meteorological Institute (www.dmi.dk) and summarized as AUC during the whole season. Titrated SPT and NAC were performed during autumn following the pollen seasons of 2012 and 2013 as described previously [9]. Titrated baseline SPT data are not available for patient 6.

We adapted the Euroflow approach to identify plasmablasts [11], and confirmed their specificity by staining with fluorochrome-conjugated grass allergen and antibody to IgE to distinguish between IgE and non-IgE producing plasmablasts. Euroflow antibodies are an established panel used for B-LCL detection. The specificity of Grass and IgE antibodies was confirmed with blood basophils from grass-sensitised patients. We calculated the number of IgE^+^, and IgE^-^ CD19^+^CD20^-^CD27^bright^CD38^bright^ plasmablasts specific for grass per ml blood using Trucount^®^ beads, at baseline before the first injection and 7 days after each allergen injection.

SMS and Rhino-conjunctivitis quality of life questionnaires (RQLQ(s)) were evaluated by calculating area under the curve (AUC) of the reported data during the pollen seasons. Data were compared using Wilcoxon matched pairs signed-rank test. Individual data and medians are displayed.

Grass pollen component specific IgE and IgG4 levels of eight available components were measured on the ISAC-chip (Thermo Fisher Scientific, Uppsala, Sweden) before starting ILIT and during autumn following the first grass pollen season after the treatment. For the comparisons in this study the values for the eight components were summed for each patient, timepoint and isotype.

Basophil sensitivity was determined five times (before treatment, after every treatment injection and after one allergen season) with Aquagen Grass allergen (ALK Hørsholm, Denmark) as described previously [9].

## Results

**Fig. 1:**
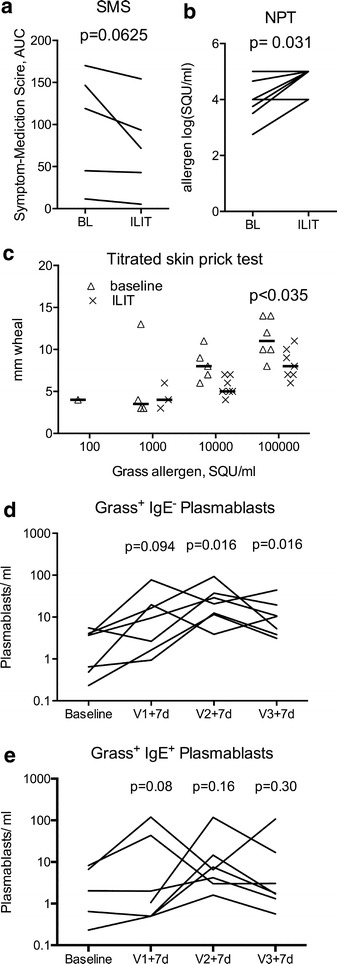
Impact of ILIT on Symptom medication score (SMS) (**a**), nasal allergen challenge (NAC) (**b**), skin prick test diameter >3 mm (**c**) and allergen specific non-IgE (**d**) and IgE (**e**) plasma cell numbers 1 week after each allergen injection (Vn + 7d). Nonparametric tests were used to evaluate treatment effect

Seven patients received three injections. One participant registered local swelling at the injection site, a second patient registered local itching and a third decrease by 19 % in PEF, that quickly normalized after inhalation of beta_2_-agonist. All these side effects occurred after the first ILIT injection. We compared combined symptom–medication scores (SMS) from the previous years´ seasons to the grass pollen season 2013 (Fig. 1a). Total grass pollen counts and length of the pollen season were comparable between the seasons of 2012 (2390 grains) and 2013 (2216 grains). SMS were reduced after ILIT (p = 0.0625). RQLQ scores from season 2013 were comparable to that of previous seasons (p = 0.26). Tolerance to grass pollen increased significantly in NAC (p = 0.031) and titrated SPT (p < 0.006) performed before ILIT and after the ensuing grass pollen season (Fig. 1b, c).

In ILIT allergen is injected directly into the lymph node to strongly stimulate the adaptive immune system at a known time and date. We hypothesized that we would be able to detect allergen-specific non-IgE plasma blasts 7 days after allergen injection into a lymph node as has been shown for tetanus vaccination [10]. The concentration of circulating plasmablasts increased after ILIT. Normalised to day 7 (100 %), there were 41 % on day 6, 102 % on day 8 and 88 % plasmablasts on day 9 after an intralymphatic allergen injection (not shown). Seven days after the first injection, we found more than a doubling of allergen specific, non-IgE plasmablasts in all patients (Fig. 1d). After the second injection we found a >fivefold increase in the allergen specific, non-IgE plasmablast concentration (p = 0.016). After the third injection, we found more than a doubling of allergen specific, non-IgE plasmablast concentration compared with the baseline visit (p = 0.016). The increase in allergen-specific, IgE^+^ plasmablasts did not reach significance at any visit (Fig. 1e), but maximal increase 7 days after any injection in total grass specific plasmablasts, non-IgE plasmablasts and IgE^+^ plasmablasts was significant (p = 0.0078). The frequency of birch-specific IgE^−^ plasmablasts in the single patient double sensitised with birch and grass did not change.

Summed IgE specific for eight grass allergens did not increase during treatment; the median at baseline was 9.3 ISU/L (IQR 0.73–69.63), and 57.91 ISU (IQR 21.53–76.66) after treatment and one season (p = 0.24). The corresponding IgG4 measurements were 0.03 ISU/L (IQR 0.0–0.96) at baseline, and 0 ISU/L (IQR 0–0) after treatment and one season (p = 0.9).

Basophil sensitivity was attenuated during subcutaneous immunotherapy, and predicted treatment success [9]. We assessed basophil sensitivity as a biomarker of ILIT at five visits; at baseline, and preceding every allergen injection and after one grass pollen season. Neither basophil reactivity nor sensitivity changed significantly as a result of ILIT (data not shown).

## Discussion

Self-reported symptom–medication scores of five patients as well as paraclinical measures of seven patients suggest that ILIT may relieve symptoms and reduce medication use, and increase the threshold at which patients respond to NAC and SPT. The three patients with the most severe symptoms reported a decrease in SMS.

In this study, a radiologist and a pulmonary consultant trained to biopsy lymph nodes identified lymph nodes and injected allergen to ensure a correct deposition of allergen in inguinal lymph nodes. We recorded 3 side effects amongst 21 allergen injections, only one of them a mild grade 1 systemic reaction. This was similar to the side effects reported from previous studies [4, 5]. A higher rate of side effects may be explained by allergen injected proximal to, rather than into lymph nodes. Injection frequency may be crucial for the clinical efficacy [7]. We adhered to allergen injection at 4-week intervals used where ILIT was successful [4, 5, 8].

Allergen specific IgE^+^ and IgE^−^ plasmablasts were detected in patient blood. The frequency of allergen specific IgE^−^ plasmablasts increased significantly 7 days after injection of allergen into an inguinal lymph node. In contrast to previous reports [12] we determined absolute concentrations between 1 and 100 plasmablasts/ml. This is a first report characterising plasmablasts arising from AIT that will have to be refined and confirmed by other methods.

In contrast to SCIT, we found no significant changes in grass pollen component specific IgE or IgG4 levels [13] or in basophil sensitivity [9]. The post ILIT samples may have been taken too soon after the grass pollen season. Alternatively, the mechanism of ILIT may be different—and possibly similar to that induced by peptide immunotherapy [14]. In both peptide allergen immunotherapy and ILIT, the allergic effector cell (mast cell and basophil granulocyte) adaptation described for SCIT [15, 16] may be circumvented, and the adaptive immune response targeted directly.

We report an open trial of ILIT that may have a large placebo effect. The primary outcome parameter, improvement of SMS, was not significantly reduced. However, allergen specific plasmablasts were induced by treatment, and objective measures of clinical improvement, NAC and SPT, improved. We are now exploring the effect of ILIT on grass pollen induced rhino-conjunctivitis in a double blind, placebo controlled trial (EUDRACT 2012-005227-33).

## Abbreviations

ILIT: intralymphatic immunotherapy
AIT: allergen immuotherapy
SMS: symptom medication score
SCIT: subcutaneous immunotherapy
NAC: nasal allergen challenge
GCP: good clinical practise
RQLQ: rhinoconjunctivitis quality of life questionnaire
PEF: peak expiratory flow
SPT: skin prick test
